# RGC-YOLO: a lightweight YOLOv8n-based detector for edge-deployable weed detection in Xinjiang cotton fields

**DOI:** 10.3389/fpls.2026.1911031

**Published:** 2026-09-10

**Authors:** Qianqian Mu, Yongke Li, Nueraili Aierken, Lei Wang, Tao Zhao, Mengli Shan, Bowen Mao, En Yu

**Affiliations:** 1 College of Computer and Information Engineering, Xinjiang Agriculture University, Urumqi, China; 2 College of Computer Science and Technology, Zhejiang University, Hangzhou, China

**Keywords:** deep learning, edge deployment, lightweight networks, precision agriculture, weed detection

## Abstract

The cotton field weed detection model must balance detection accuracy, model size, and inference efficiency when deployed at the edge. This study constructed a dataset comprising four types of weeds based on field images collected from cotton fields in Xinjiang and proposed a lightweight detection model, RGC-YOLO, based on YOLOv8n. The model incorporates R-UIB modules into the deep layers of the backbone network to reduce the number of parameters and computational load during the high-channel feature extraction stage. A G-C2f module is introduced at the P5 feature fusion point in the neck network to improve the efficiency of deep semantic feature fusion. Additionally, a DIoU loss function is employed to strengthen the constraints on bounding box localization. The results show that, compared to YOLOv8n, RGC-YOLO achieves a 2.65% improvement in the mAP@0.75, while reducing the number of parameters and GFLOPs by 35.22% and 15.37%, respectively. This indicates that the model effectively reduces complexity while enhancing high-IoU localization performance. Eigen-CAM analysis further demonstrates that RGC-YOLO produces more concentrated feature responses in weed target regions and reduces irrelevant background activations. On the Jetson AGX Orin platform, the FP16 TensorRT engine for RGC-YOLO achieves high throughput and a compact footprint, demonstrating that its lightweight design translates into benefits for edge deployment. External validation on a sesame-field binary crop–weed dataset and the 2SeasonWeedDet8 multi-class weed dataset further confirms that RGC-YOLO maintains comparable detection performance while preserving its lightweight advantage across different agricultural scenarios.

## 1 Introduction

Xinjiang is China’s primary source of cotton, accounting for approximately 80% of the country’s total cotton output (Zhou et al., 2025). According to reports, weeds and cotton compete for water, nutrients, sunlight, and growing space throughout their growth cycles, and weeds can serve as hosts for pests and diseases, thereby reducing cotton yields and increasing field management costs (Wang et al., 2025; Zhang et al., 2025). At present, weed control primarily relies on manual weeding, mechanical weeding, and the application of herbicides. However, these methods not only result in high production costs but also lead to environmental pollution and the emergence of herbicide-resistant weeds (Vignesh et al., 2025). Therefore, the development of precise, efficient, and environmentally friendly weed control technologies is of great importance.

With the advancement of computer vision technology, object detection algorithms are increasingly being applied to weed identification and localization. These algorithms provide information on weed types and locations, enabling targeted spraying and mechanical weed control, thereby helping to reduce production costs and improve the precision of weed control operations (Gao et al., 2025; Singh et al., 2025). Deep learning-based object detection algorithms can generally be categorized into two-stage and single-stage detection methods. Compared to two-stage detection methods, single-stage detection methods can directly perform object classification and location regression, offering higher inference efficiency. In particular, the single-stage YOLO series strikes a good balance between detection accuracy and inference speed and has been widely adopted for weed detection in agricultural fields. For cotton-field weed detection, Dang et al. (2023) constructed the multi-class CottonWeedDet12 dataset and systematically evaluated different YOLO detectors, demonstrating the applicability of YOLO models for weed identification and localization under natural cotton-field conditions. To address dense weed distribution, partial occlusion, and missed detection of small targets in complex cotton fields, Zheng et al. (2024b) enhanced multi-scale feature extraction, introduced an additional small-object detection layer, and optimized feature fusion and post-processing. Ren et al. (2024) further integrated local and global feature information and improved the feature reconstruction process to enhance weed representation under target-scale variation and leaf occlusion. In other crop–weed detection scenarios, Sonawane et al (Sonawane and Patil, 2025), Chen et al. (2022), Deng et al. (2024), and Wang et al. (2022) introduced attention mechanisms and optimized feature fusion strategies to improve detection performance under complex field conditions. Ma et al. (2025) combined attention-based feature enhancement with bounding-box regression optimization to improve crop–weed discrimination and target localization. Yang et al. (2025) and Peng et al. (2025) further explored lightweight architectural optimization to improve weed detection performance while reducing computational complexity. Overall, existing YOLO object detection methods have improved weed detection performance in complex field environments through techniques such as attention mechanisms, multi-scale feature fusion, and bounding box regression optimization. However, while some improvement methods enhance the model’s ability to represent features, they also increase its complexity, making it difficult to balance detection performance with the requirements for deployment on edge devices.

In response to the challenges posed by high model complexity and limited deployment capabilities on edge devices, researchers have increasingly incorporated lightweight design into weed detection models in recent years. Fan et al. (2024) proposed YOLO-WDNet, which reduces computational overhead by optimizing the network architecture to meet the real-time detection requirements of precision pesticide application robots in cotton fields. Karim et al. (2024) developed a lightweight model for detecting weeds in cotton fields based on YOLOv8 and validated the model’s feasibility for weed identification and localization tasks on edge devices. Zheng et al. (2024a) introduced a lightweight backbone network and attention mechanisms to improve the performance of cotton field weed detection while reducing model complexity. Wang et al. (2025) further reduced the number of model parameters and computational complexity by optimizing the network architecture of YOLOv8n. Lu et al. (2025) proposed Star-YOLO, which improves the model’s detection efficiency through a lightweight architectural design and optimized feature fusion. More recently, Wang et al. (2026) deployed a YOLOv10-based weed detection and segmentation framework on the Jetson Xavier NX and evaluated it in natural winter-wheat fields using a UAV platform. Collectively, these studies show that current lightweight weed detectors adopt strategies such as lightweight backbone reconstruction, attention-based feature enhancement, feature-fusion optimization, and lightweight detection-head design to balance detection performance and computational efficiency. However, network structure compression may also weaken the model’s ability to extract and integrate features. In complex cotton field environments—particularly those with significant variations in weed size, strong background interference, and noticeable plant occlusion—issues such as reduced detection performance and inaccurate bounding box localization are still likely to occur. Thus, how to reduce model complexity while maintaining effective feature representation capabilities and further improving the localization performance of weed targets remains a key challenge in weed detection in cotton fields.

Beyond conventional object detection methods designed for predefined categories, recent advances in vision–language models (VLMs) have provided new possibilities for visual understanding and semantic interaction in precision agriculture. By jointly modeling visual information and textual semantics, VLMs can support cross-modal retrieval, zero-shot recognition, visual question answering, and image-based reasoning, thereby reducing their dependence on task-specific annotations and predefined categories (Haghighat et al., 2025; Zhu et al., 2025). Recent studies have extended VLM-based agricultural analysis from visual recognition to multimodal diagnosis and quality assessment. Zhang et al. (2024) developed a visual large language model for wheat disease diagnosis under natural field conditions, whereas Huang et al. (2025) systematically evaluated and deployed large VLMs for multi-dimensional fruit quality assessment. Nguyen Quoc et al. (2025) further developed an agricultural vision–language foundation model and demonstrated its potential for zero-shot and few-shot leaf disease identification. More directly related to weed management, Nasir et al. (2026) evaluated multiple VLMs for zero-shot weed detection, spatial localization, and visual reasoning using UAV-based agricultural images. These studies demonstrate the potential of VLMs for recognizing unseen agricultural targets and interpreting complex field scenes. However, their performance remains affected by agricultural domain differences, fine-grained visual ambiguity, spatial localization reliability, and high computational requirements. Therefore, VLMs represent a promising direction for extending weed detection toward open-category recognition and visual reasoning, whereas efficient supervised detectors remain important for real-time localization on resource-constrained edge devices.

To address the challenge of balancing detection performance and computational overhead in edge-deployed cotton-field weed detection, this study proposes RGC-YOLO, a lightweight weed detection model based on YOLOv8n. RGC-YOLO adopts a selective lightweight optimization strategy focused primarily on deep, high-channel feature processing, employing R-UIB to reduce the number of parameters and computational overhead involved in feature extraction at deep layers of the backbone network. During the feature fusion stage, G-C2f is positioned at the P5 semantic fusion node to reduce redundant computations in high-level feature transformations, whilst preserving the transmission and fusion of target information at different scales from P3 and P4. To minimize the potential impact of structural compression on bounding box localization, DIoU is utilized to strengthen the spatial constraints between predicted and ground-truth boxes. This work aims to reduce the model size whilst maintaining the ability to represent and localize multi-scale weed features, thereby achieving a more reasonable balance between detection performance and model complexity.

## 2 Materials and methods

### 2.1 Weed image dataset

To establish a dataset for weed detection in Xinjiang cotton fields, this study conducted field image acquisition from May to July in experimental cotton fields located in Qiaogetireke Village, Shaya County, and Hua Xing Farm, Changji City, Xinjiang. The sampling period was divided into 18 sampling phases, with approximately 3 to 5 days between adjacent phases. Data collection covered weather conditions such as sunny, cloudy, and overcast skies, as well as different times of day, and included field images taken from various heights and angles. The data was collected using portable imaging devices such as smartphones.

**Figure 1:**
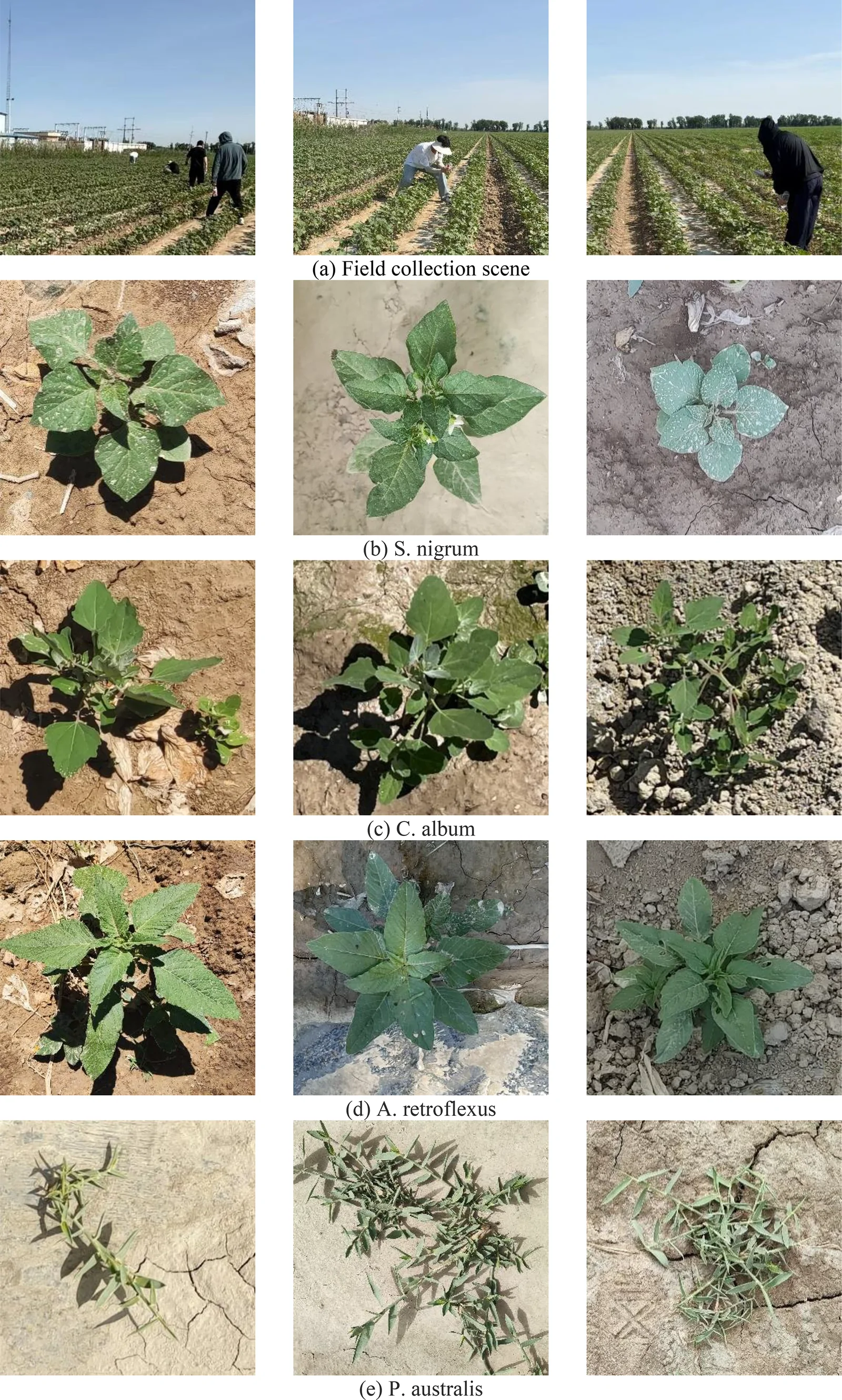
Field collection scene and representative samples of the four target weed species. **(a)** Field collection scene. **(b)** S. nigrum. **(c)** C. album. **(d)** A. retroflexus. **(e)** P. australis.

The dataset focuses on four common weed species found in cotton fields in Xinjiang: Solanum nigrum L.(S. nigrum), Chenopodium album L.(C. album), Amaranthus retroflexus L.(A. retroflexus) and Phragmites australis (Cav.) Trin. ex Steud.(P. australis). After image screening and quality checks, a total of 3,162 field images were retained. **Figure 1** shows the field collection site and representative samples of the four weed species.

In this study, the X-AnyLabeling software was used to annotate weed targets and to check for issues such as missing annotation files, abnormal category IDs, out-of-bounds coordinates, and formatting errors. To enhance the dataset’s ability to represent real-world field conditions, images that have been manually verified as free of target weeds retain their empty annotation files and are used as negative background samples in model training and evaluation. Target instances that remain recognizable at the edges of the image are also retained to reflect situations where targets approach the image boundaries during actual data collection.

After completing image screening, object annotation, and quality checks, this study divided the dataset into subsets. To mitigate the risk of information leakage caused by the distribution of consecutively captured images or images of similar scenes across subsets, a group-level partitioning strategy is adopted, using image groups formed from the same continuous scene as the smallest partitioning unit for group-level allocation. Based on this, we take into account factors such as the proportion of background negative samples, instance density, target size distribution, edge target distribution, and the proportion of samples from different collection sites to ensure that the training, validation, and test sets maintain relatively consistent data distributions. Ultimately, the training set, validation set, and test set accounted for approximately 72.6%, 13.3%, and 14.0%, respectively. The proportions of background and negative sample images were approximately 14.5%, 14.5%, and 16.7%, respectively. Detailed information about the dataset is shown in **Table 1**.

**Table 1:** Dataset split and distribution of target instances by category.

Data	Images	Positive images	Background images	Scene groups	*Instances*	*S. nigrum*	*C. album*	*A. retroflexus*	*P. australis*
Training set	2297	1964	333	120	3733	1979	727	491	536
Validation set	422	361	61	32	827	466	191	88	82
Test set	443	369	74	20	821	462	164	112	83
Total	3162	2694	468	172	5381	2907	1082	691	701

As shown in **Table 1**, a total of 5,381 weed instances were annotated in this study, indicating that the dataset does not consist solely of single-object images. A total of 1,061 images contained two or more weed instances, accounting for 33.55% of all images and 39.38% of the positive samples. These multi-object images contain a total of 3,748 weed instances, accounting for 69.65% of all annotated instances. Furthermore, multi-object images account for 47.15% of the positive samples in the test set, enabling the model to be evaluated in complex scenes containing multiple weed targets.

**Figure 2:**
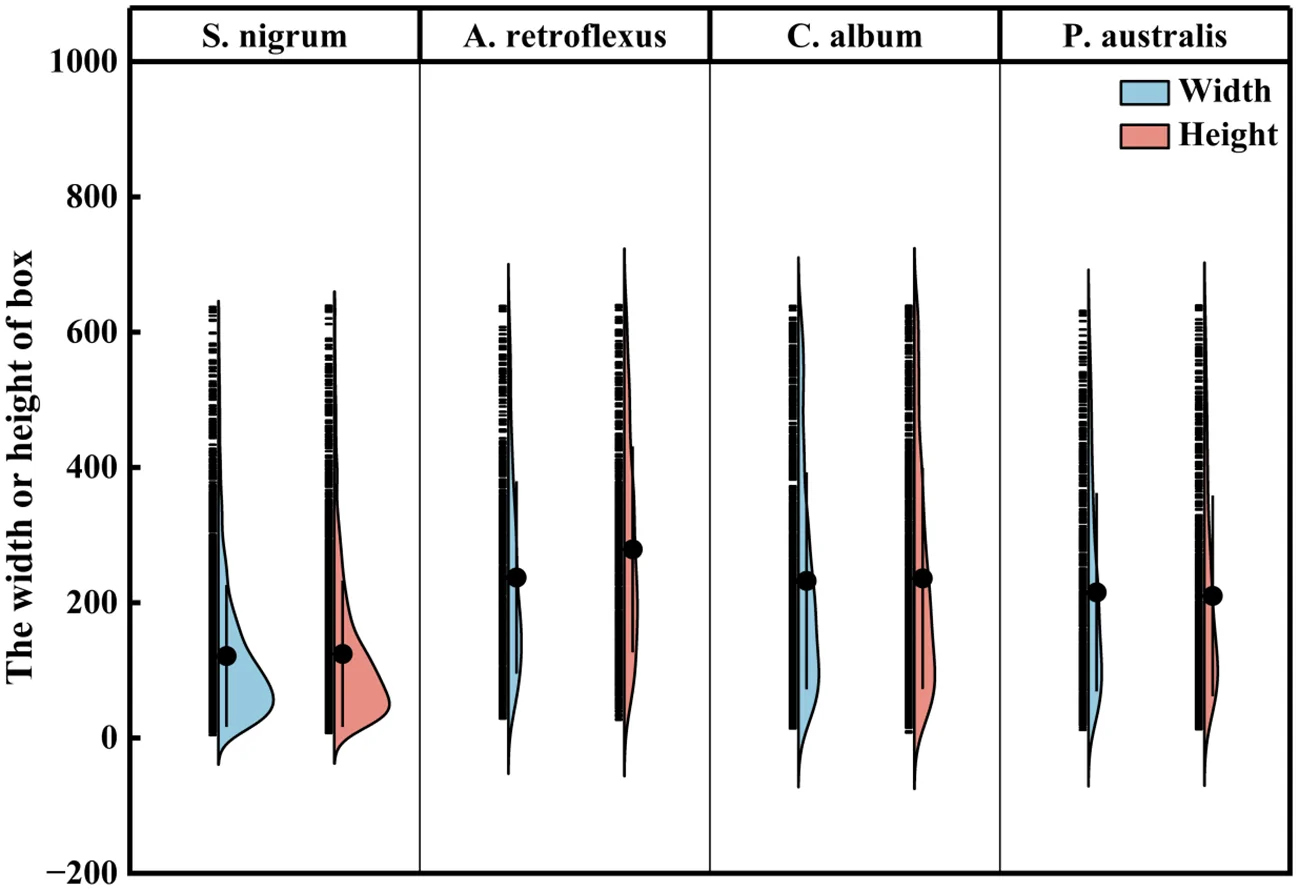
Distribution of width and height of bounding boxes for four types of weeds.

**Figure 2** shows the distribution of the width and height of the bounding boxes for the four types of weeds in the dataset. The statistical results are expressed in pixels and are calculated based on a standard input size of 640 × 640. Specifically, the bounding boxes for S. nigrum are generally smaller, with median widths and heights of approximately 90 and 92 pixels, respectively, and a median bounding box area-to-image-area ratio of approximately 2.0%. In contrast, the bounding box dimensions for C. album, A. retroflexus, and P. australis are generally larger. Among them, A. retroflexus had the highest median height, at approximately 255 pixels, and the median ratio of the bounding box area to the image area was approximately 12.4%. The median width and height of C. album are approximately 195 and 200 pixels, respectively, while those of P. australis are approximately 185 and 165 pixels, respectively. The above statistical results indicate that there are significant scale differences among the various categories of weed targets in the dataset, which places high demands on the model’s ability to characterize features across multiple scales.

### 2.2 Construction of the RGC-YOLO model

YOLOv8n is the nano-sized model in the YOLOv8 series, characterized by its small parameter size and low computational cost (Varghese and Sambath, 2024). In recent years, YOLOv8n has been widely adopted as a baseline model for lightweight modifications in agricultural vision research and has been applied to tasks such as weed detection and real-time edge-based recognition (Jiang et al., 2024; Ma et al., 2024; Farhan et al., 2025; Feng et al., 2025; Li et al., 2025; Sun et al., 2025). Given the complex background noise and variations in target size present in the dataset used in this study, and considering that deployment at the edges of cotton fields places high demands on model size and computational overhead, YOLOv8n was selected as the baseline model in this study.

**Figure 3:**
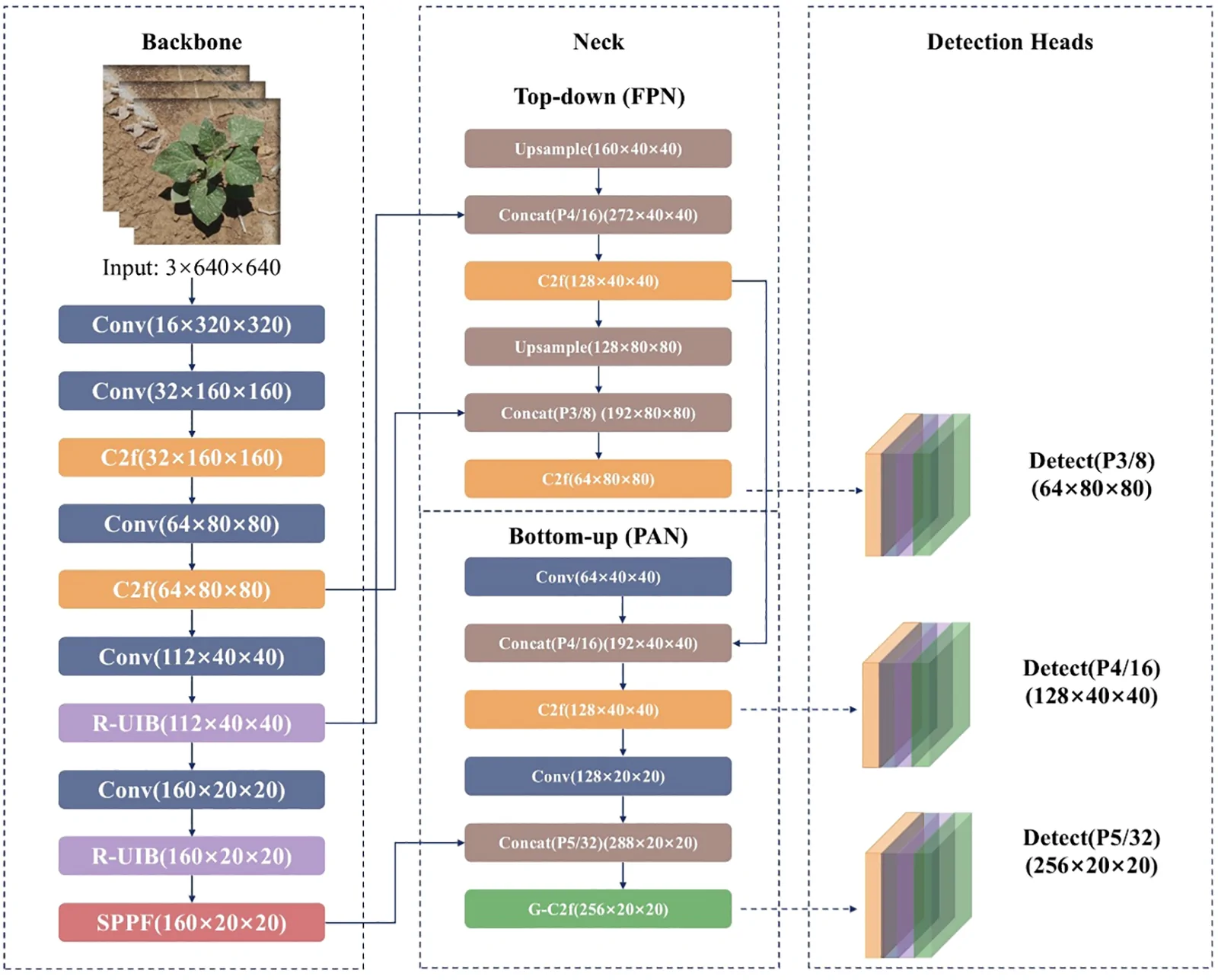
Overall network architecture of RGC-YOLO.

Based on YOLOv8n, this study develops RGC-YOLO by focusing on three key areas: lightweight structural reconstruction, optimization of deep feature fusion, and improvement of bounding box localization performance. Specifically, R-UIB modules are introduced deep within the backbone network to reduce the number of model parameters and computational overhead while preserving as many effective deep semantic features as possible. The G-C2f module is introduced at the P5 feature fusion layer of the neck network to optimize deep semantic feature fusion and reduce redundant computations during high-dimensional feature transformations. In bounding box regression, the DIoU loss is employed to improve the spatial alignment between predicted and ground-truth bounding boxes by introducing a constraint on the distance between their center points, thereby enhancing bounding box localization performance. The overall network architecture of RGC-YOLO is shown in **Figure 3**.

#### 2.2.1 R-UIB module

In the YOLOv8n backbone network, the P4/16 and P5/32 layers are located in the deeper stages of feature extraction, they have relatively high channel dimensions and offer some potential for structural simplification. Given that the shallow layers still bear the brunt of conveying spatial details, this study retains the shallow network architecture and introduces R-UIB modules at the P4/16 and P5/32 stages. At the same time, we apply appropriate compression to the deep output channels at the network level to reduce the number of parameters and computational overhead in the deep backbone network, while minimizing the impact of this structural simplification on effective feature extraction.

**Figure 4:**
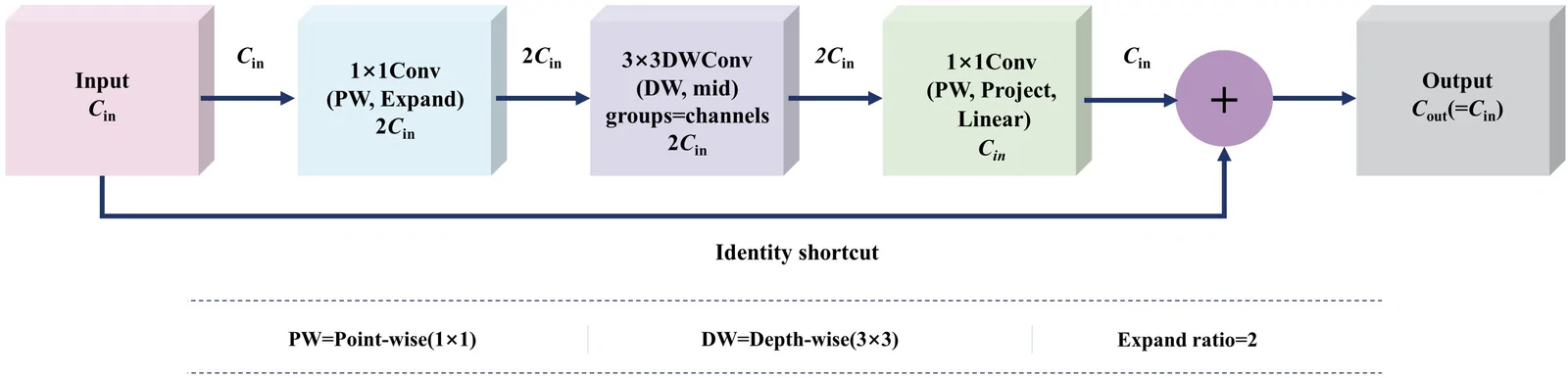
Detailed structure of the R-UIB module.

The R-UIB module draws on the lightweight design philosophy of the Universal Inverted Bottleneck (UIB) architecture from MobileNetV4 and is restructured based on the basic paths of the inverted bottleneck architecture (Sandler et al., 2018; Qin et al., 2024). To balance computational cost and feature representational capacity, this study sets the channel expansion ratio to 2 and employs a lightweight feature transformation path consisting of point-wise convolution, deep convolution, linear projection, and an identity residual connection. Specifically, pointwise convolution is used for channel expansion and projection, deep convolution is used to extract local spatial information with lower computational cost, and identity residual connections are used to facilitate feature propagation. Through the above design, the R-UIB module aims to reduce the structural complexity of deep backbone networks and minimize the impact of lightweight processing on deep semantic feature representations, thereby providing more compact deep feature inputs for subsequent neck networks. The specific structure of the R-UIB module is shown in **Figure 4**.

#### 2.2.2 G-C2f module

Background noise and differences in target scale in complex cotton field environments require the neck network to retain effective multiscale feature fusion capabilities even after being streamlined. In the YOLOv8n neck network, the P5 layer is primarily responsible for integrating deep semantic information, it has a relatively high channel dimension and offers some room for structural optimization. In contrast, the P3 and P4 layers still retain a significant amount of spatial detail and mid-level feature information. If extensive lightweight replacements are made to the neck network, it may affect the propagation of multiscale features. Therefore, this study retains the standard C2f architecture at the P3 and P4 levels, introducing the G-C2f module only at the feature fusion point of the P5 scale.

The G-C2f module draws on GhostNet’s approach to lightweight feature generation (Han et al., 2020). GhostNet generates supplementary feature maps using a small number of intrinsic feature maps and low-cost operations, thereby reducing redundant computations in the standard convolution process. While retaining the feature splitting and concatenation fusion path of C2f, this study employs GhostBottleneck to perform a lightweight reconstruction of the internal feature transformation branches.

**Figure 5:**
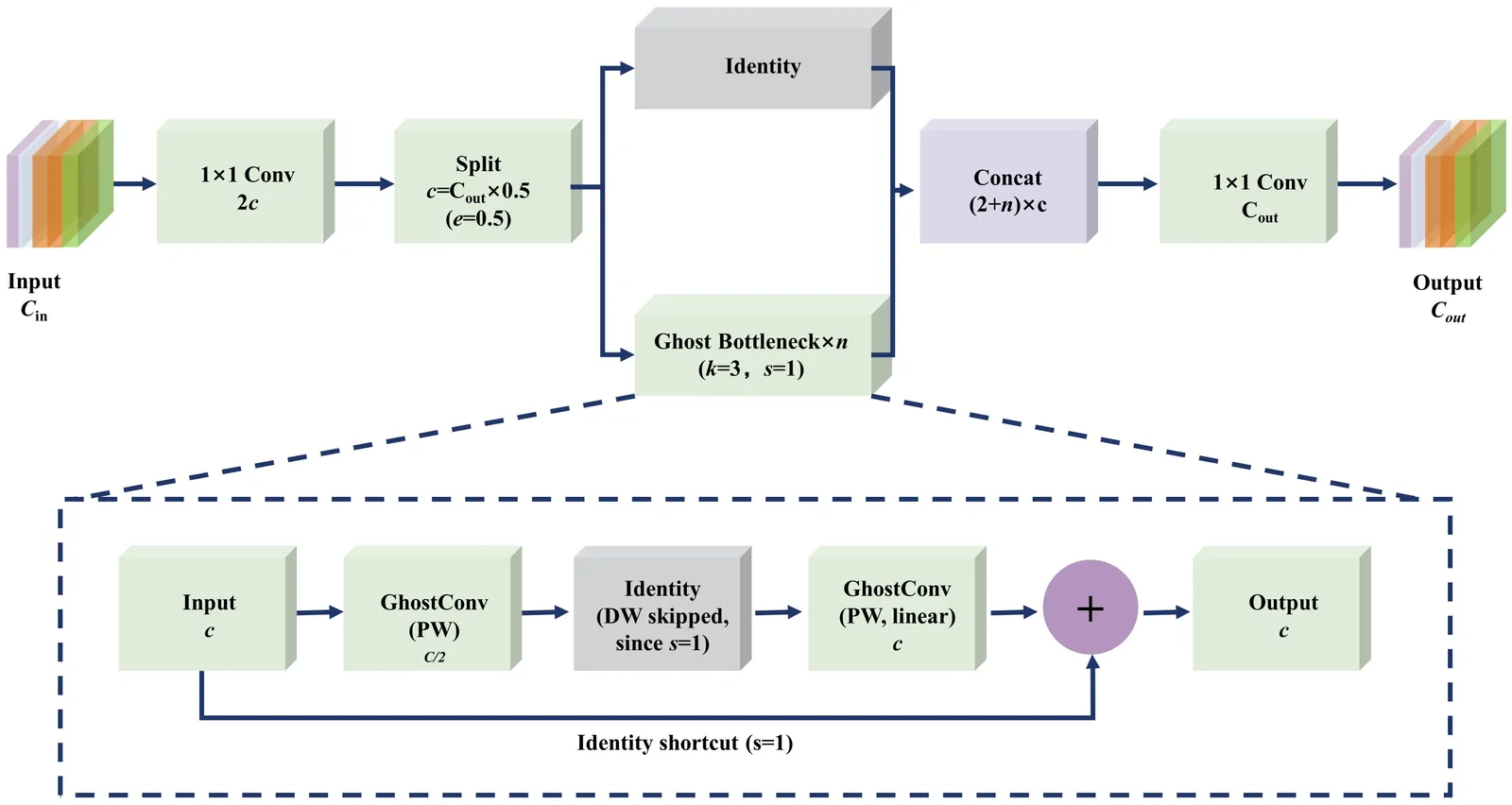
Detailed structure of the G-C2f module.

Through the above design, the G-C2f module aims to reduce redundant computations during the fusion of deep semantic features at the P5 level, while maintaining effective deep feature fusion capabilities as much as possible while controlling model complexity. This mitigates the impact of network lightweighting on feature representation in complex cotton field environments. The specific structure of the G-C2f module is shown in **Figure 5**.

#### 2.2.3 DIoU loss function

Background noise, variations in target size, and plant occlusion in complex cotton field environments make it difficult to perform bounding box regression on weed targets. To strengthen the spatial constraints between the predicted bounding box and the ground-truth bounding box and improve the localization performance of weed targets, this study employs Distance-IoU (DIoU) as the IoU loss term in bounding box regression (Zheng et al., 2020).

DIoU introduces a constraint on the distance between the center points of the predicted bounding box and the ground-truth bounding box, based on the IoU overlap. The formula is as follows:

(1) $$DIoU=IoU-\frac{\rho^{2}(b,b^{gt})}{c^{2}},\;L_{DIoU}=1-DIoU$$

**Figure 6:**
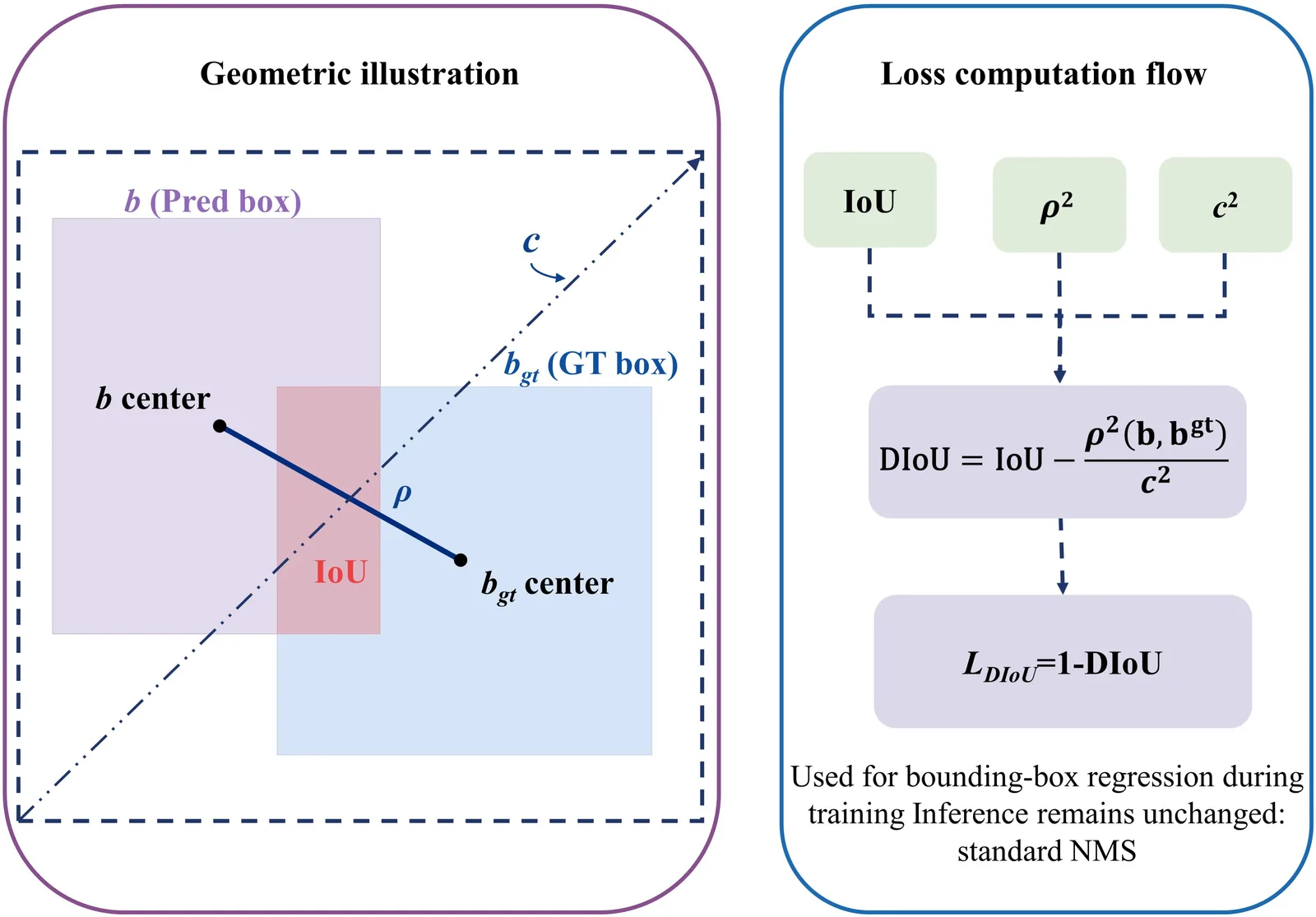
Geometric relationships and computational principles of the DIoU loss function.

Among these, b and $b^{gt}$where represents the center coordinates of the predicted bounding box and the ground-truth bounding box, $\left(b,b^{gt}\right)$ denotes the Euclidean distance between the centers of the two boxes, and c denotes the length of the diagonal of the smallest bounding rectangle that encloses both the predicted box and the ground-truth box. The geometric relationships and calculation principles of DIoU are shown in **Figure 6**.

### 2.3 Training settings and hyperparameters

To ensure the comparability of results across different experiments, all experiments were conducted on the same hardware platform. The experimental platform is equipped with an Intel Core i9-14900HX processor, 32GB of RAM, and an NVIDIA GeForce RTX 4060 Laptop GPU (8GB VRAM), and runs on Windows 11. The main software environment consisted of Python 3.9.24, PyTorch 2.5.1, and CUDA 12.1.

To minimize the impact of training randomness on the experimental results, both the core model comparison experiments and the ablation experiments were conducted three times with independent training runs using different random seeds. For the YOLO-based models and the associated ablation experiments, the same random seeds (43, 48, and 50) were used for the three independent runs. After each training run, the model weights used for evaluation were determined based on the validation set results, and the evaluation metrics were calculated on the same test set. For experiments conducted with three independent runs, the reported aggregate model-level metrics are presented as the mean and standard deviation (SD). During training, all models employed the same online data augmentation strategy. The main training hyperparameters are shown in **Table 2**.

**Table 2:** Training hyperparameter settings.

Hyperparameter name	Value/setting
Image Size	640×640
Batch size	16
Epochs	200
Optimizer	SGD
Initial learning rate (lr0)	1×10^-2^
Final learning rate factor (lrf)	1×10^-2^
Momentum	0.937
Weight decay	5×10^-4^
Patience	50
Close mosaic epoch	10

lr0 denotes the initial learning rate, and lrf denotes the final learning rate factor. The final learning rate is calculated as lr0 × lrf.

### 2.4 Evaluation metrics

To comprehensively evaluate the model’s detection performance and level of optimization in the task of weed detection in cotton fields, this study uses precision (P), recall (R), F1-score (F1), average precision (AP) mAP@0.5, mAP@0.75 and mAP@0.5:0.95 as performance evaluation metrics for detection, and uses the number of parameters and floating-point operations (GFLOPs) to evaluate model complexity.

Among these, precision refers to the proportion of correctly detected objects out of all predicted objects, recall refers to the proportion of correctly detected objects out of all ground-truth objects, and the F1 score is the harmonic mean of precision and recall. AP refers to the area under the precision–recall curve for a single category, while mAP refers to the average of the AP values across all categories. mAP@0.5 and mAP@0.75 represent the mean average precision values for IoU thresholds of 0.5 and 0.75, respectively, while mAP@0.5:0.95 represents the average results obtained by varying the IoU threshold in the range of 0.5 to 0.95 in 0.05 increments. The number of parameters reflects the model’s scale, while GFLOPs measure the theoretical computational complexity of a single inference run. The formulas for calculating the evaluation metrics are given in Equations 1–6:

(2) $$P=\frac{TP}{TP+FP}$$

(3) $$R=\frac{TP}{TP+FN}$$

(4) $$F1=\frac{2PR}{P+R}$$

(5) $$AP_{i}=\int_{0}^{1}P_{i}(R_{i})dR_{i}$$

(6) $$mAP=\frac{1}{n}\sum_{i=1}^{n}AP_{i}$$

In the above equations, *TP*, *FP* and *FN* signify the number of correctly detected targets, the number of false positives, and the number of false negatives, respectively. *P_i_*(*r*) is the precision corresponding to a recall of r on the precision–recall curve for class *i*, *AP_i_* denotes the average precision for class i, and n represents the number of target classes. Since the dataset used in this study contains four weed classes, *n* = 4.

Further, in order to quantitatively evaluate the spatial alignment consistency between the Eigen-CAM response maps and the actual weed areas, this study employs the Pointing Game (PG) accuracy and the Energy-Based Pointing Game (EBPG) for evaluating object detection-based map localization (Yamauchi, 2024; Panda et al., 2025). In this approach, PG evaluates the localization consistency between the response peak and the annotated target by determining whether the position of the maximum response in the response map lies within the true target region (Zhang et al., 2018). Meanwhile, EBPG calculates the proportion of response energy within the true target region relative to the total response energy across the entire image, and is used to measure the degree to which the model’s response is concentrated within the target region (Wang et al., 2020).

For the positive sample image number *i*, let $A_{i}(p)$ denote the normalized Eigen-CAM response value at pixel position *p*, $M_{i}$ represent the union of all ground-truth bounding boxes in the image, $\Omega_{i}$ represent the entire image area, and *N* represent the number of positive sample images included in the evaluation. For images containing multiple weed targets, all ground-truth bounding boxes are merged into a single union region, and overlapping areas between bounding boxes are counted only once. The formula for EBPG is given in Equation 7:

(7) $$EBPG=\frac{1}{N}\sum_{i=1}^{N}\frac{\sum_{p\in M_{i}}A_{i}(p)}{\sum_{p\in {\text{Ω}}_{i}}A_{i}(p)+\epsilon }$$

Where ϵ is a very small positive number chosen to avoid a zero denominator. The higher the EBPG value, the greater the concentration of the Eigen-CAM response energy within the region of the true target. $S_{i}$ represents the set comprising the positions of all pixels with the highest response within image, as defined in Equation 8:

(8) $$S_{i}=\{p\in {\text{Ω}}_{i}|A_{i}(p)=\underset{q\in \Omega_{i}}{\max}A_{i}(q)\}$$

The formula for calculating the PG accuracy rate is given in Equation 9:

(9) $$PG_{accuracy}=\frac{1}{N}\sum_{i=1}^{N}I(S_{i}\cap M_{i}\neq \emptyset )$$

In the formula, I(·) represents the indicator function. I(·) hit is recorded when the pixel with the maximum response lies within any of the ground-truth bounding boxes; otherwise, it is recorded as a miss. Where there are multiple pixels in the response map with the same maximum response value, a hit is recorded provided that at least one of them lies within the ground-truth target area. A higher PG accuracy indicates better spatial consistency between the response peak and the ground-truth weed area. Both EBPG and PG accuracies are expressed as percentages.

## 3 Results and discussion

### 3.1 Performance comparison with representative object detection models

To evaluate the detection performance and computational efficiency of RGC-YOLO in complex cotton field environments, this study selected Faster R-CNN (Ren et al., 2016; Cui et al., 2024), RetinaNet (Lin et al., 2017b; Peng et al., 2022), RT-DETR-R18 (Zhao et al., 2024), YOLOv5n (Mohanty et al., 2025; Sapkota et al., 2025), YOLOv8n (Ma et al., 2025; Sapkota et al., 2025), YOLO11n (Yue and Zhao, 2025) and YOLO12n (Yang et al., 2025; Tian et al., 2026), Star-YOLO (Lu et al., 2025) and PD-YOLO (Li et al., 2025) as comparison models. Faster R-CNN and RetinaNet represent typical two-stage and single-stage object detection methods, respectively. RT-DETR-R18, meanwhile, serves as a Transformer-based object detection model. YOLOv5n, YOLOv8n, YOLO11n and YOLO12n are used to compare the performance differences among various lightweight YOLO models in the task of detecting weeds in cotton fields. Star-YOLO and PD-YOLO, as recently proposed weed detection models, are used to further evaluate the competitiveness of RGC-YOLO relative to models designed for the task of detecting weeds in agricultural fields. **Table 3** shows the detection performance and computational complexity of each model.

**Table 3:** The performance comparison of different object detection models.

Model	P (%)	R (%)	mAP@0.5 (%)	mAP@0.75 (%)	mAP@0.5:0.95 (%)	Params (M)	GFLOPs
Faster R-CNN	84.82 ± 2.35	75.08 ± 1.15	80.49± 0.54	70.12± 1.30	61.88± 0.16	41.36	87.07
RetinaNet	83.82± 0.96	76.42± 1.46	82.29± 0.84	73.82± 0.53	65.84± 0.06	36.39	78.05
RT-DETR-R18	82.22± 1.49	72.33± 1.76	76.53± 1.36	68.50± 1.05	62.51± 1.13	29.75	90.60
YOLOv5n	84.30± 1.93	78.97± 1.75	85.03± 1.10	77.08± 0.76	71.81± 0.73	2.51	7.18
YOLOv8n	82.37± 2.60	77.86± 1.05	84.20± 0.76	75.87± 1.37	70.79± 1.32	3.01	8.20
YOLO11n	84.97± 2.13	75.97± 0.82	84.92± 0.73	79.24± 0.75	72.98± 0.63	2.59	6.44
YOLO12n	83.99± 0.87	80.33± 2.42	85.54± 0.41	78.37± 0.35	73.36± 0.40	2.57	6.48
Star-YOLO	79.83± 3.28	73.45± 1.22	80.56± 1.03	72.27± 1.17	65.74± 1.04	1.29	4.51
PD-YOLO	85.50± 1.32	78.24± 1.55	85.03± 0.38	77.39± 0.53	71.72± 0.30	3.49	9.17
RGC-YOLO	85.83± 0.60	78.44± 0.23	85.51± 1.14	78.52± 0.51	72.42± 0.90	1.95	6.94

As shown in **Table 3**, Faster R-CNN, RetinaNet and RT-DETR-R18 demonstrate relatively limited detection performance in terms of high IoU on the current cotton field weed dataset; furthermore, their number of parameters and GFLOPs are significantly higher than those of the lightweight YOLO model, and they do not demonstrate the complexity advantages required for edge deployment. In contrast, the YOLO series of models generally exhibit better detection performance and model scalability. RGC-YOLO achieved a precision of 85.83%, the highest among all compared models. Compared to the baseline model YOLOv8n, RGC-YOLO achieved a 3.46% increase in precision and a 1.31% increase in mAP@0.5, indicating that RGC-YOLO demonstrates better false positive suppression while maintaining its weed detection capability. Under evaluation metrics with higher IoU thresholds, RGC-YOLO achieved a mAP@0.75 of 78.52% and a mAP@0.5:0.95 of 72.42%, representing improvements of 2.65% and 1.63%, respectively, compared to YOLOv8n. This indicates that the improvement in the model is not only due to an increase in the number of detected targets, but is also reflected in more accurate target localization. These results indicate that the improvement of RGC-YOLO was not only reflected in the number of detected targets, but also in the spatial alignment accuracy of predicted bounding boxes under stricter localization requirements. Furthermore, compared to YOLOv8n, RGC-YOLO reduces the number of parameters and GFLOPs by 35.22% and 15.37%, respectively, achieving a dual optimization of improved detection performance and reduced theoretical computational overhead. This is attributable to the selective lightweight optimization strategy adopted by RGC-YOLO, which focuses on the deep-layer, high-channel feature processing and high-level semantic fusion stages rather than extensively replacing the feature extraction architecture, thereby enabling the preservation of multi-scale feature propagation capabilities whilst reducing redundant computations (Fan et al., 2024). Additionally, the improvement in the IoU metric is primarily attributable to the enhanced localization constraints during the bounding box regression process. This is because the regression optimization method for IoU introduces additional geometric constraints between the predicted box and the ground-truth box, thereby improving the spatial matching accuracy of the predicted box under strict overlap requirements (Wang et al., 2024). In summary, RGC-YOLO enhances detection performance through more efficient feature processing and localization optimization, rather than by increasing the complexity of the network.

This study compared the AP by class between the baseline model YOLOv8n and RGC-YOLO, thereby further analyzing the detection performance of RGC-YOLO across different weed classes; the results are shown in **Table 4**.

**Table 4:** Class-wise AP comparison between YOLOv8n and RGC-YOLO.

Weed class	AP@0.5 (%)		AP@0.75 (%)		AP@0.5:0.95 (%)	
	YOLOv8n	RGC-YOLO	YOLOv8n	RGC-YOLO	YOLOv8n	RGC-YOLO
*S. nigrum*	91.63	92.61	87.12	87.93	81.42	82.37
*C. album*	87.17	87.77	79.42	81.49	75.10	76.00
*A. retroflexus*	76.68	78.62	72.52	74.22	65.80	68.55
*P. australis*	81.30	83.06	64.43	70.44	60.83	62.76
Mean (mAP)	84.20	85.51	75.87	78.52	70.79	72.42

The results in **Table 4** show that RGC-YOLO outperforms the baseline model, YOLOv8n, across all four weed classes in terms of AP@0.5, AP@0.75 and AP@0.5:0.95, indicating that the improvement in overall detection performance is not attributable to any single class. Both models achieved high AP values for the S. nigrum class, whilst RGC-YOLO further improved detection performance for this class. RGC-YOLO also demonstrated varying degrees of improvement for categories where the original AP was relatively low. In particular, the AP@0.75 of P. australis increased by 6.01%, whilst that of A. retroflexus of AP@0.5:0.95 increased by 2.75%.

**Figure 7:**
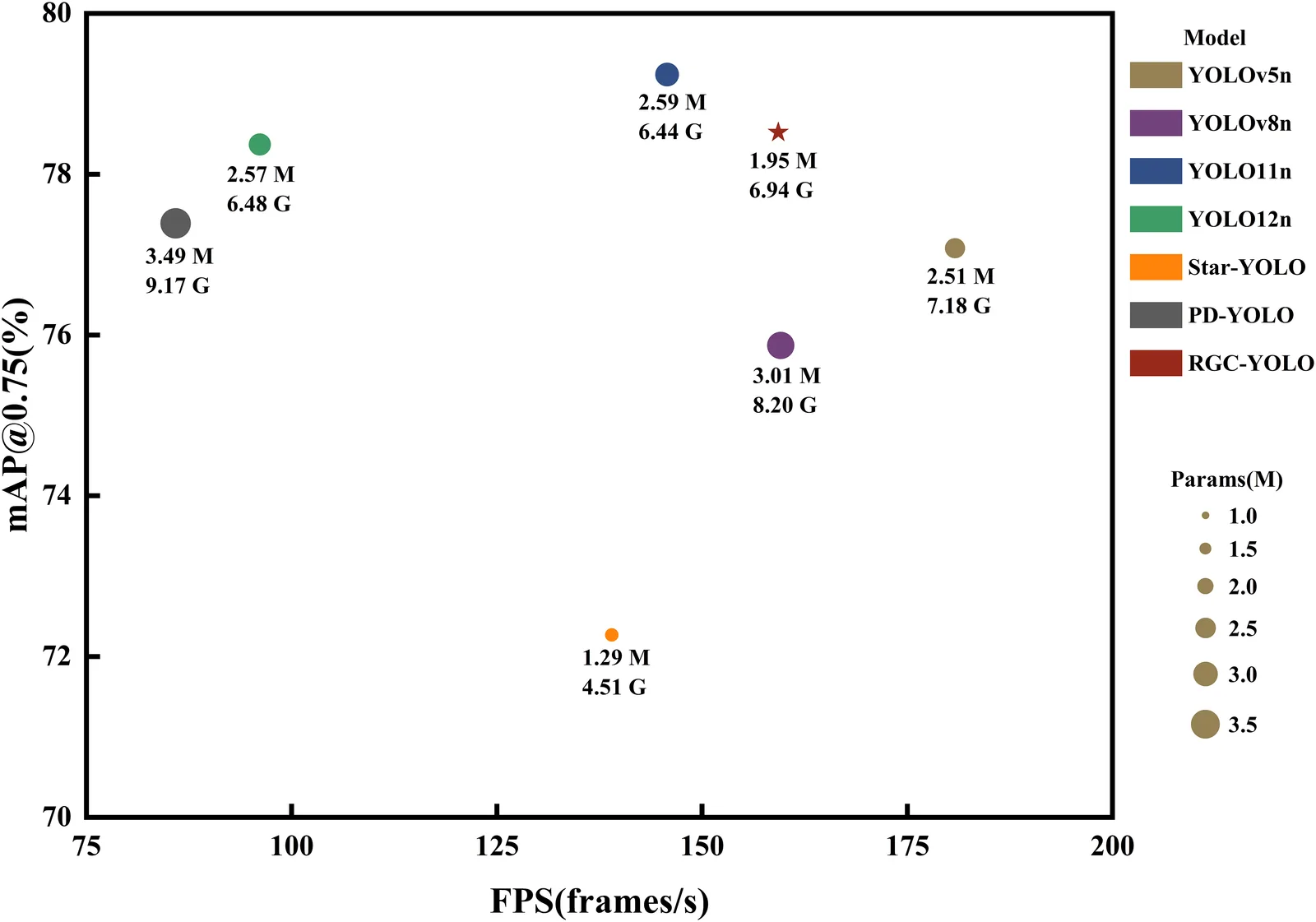
Comparison of high IoU detection accuracy, inference speed, and model complexity in the YOLO model.

To visually illustrate the trade-offs among high-IoU localization performance, inference efficiency, and model complexity of different YOLO models, this study tested the average forward inference speed of each model under the same hardware environment, with an input size of 640 × 640, FP32 precision, and single-image input (batch size = 1). An FPS–mAP@0.75 bubble plot was then generated, as shown in **Figure 7**. To ensure a fair comparison, only YOLO models evaluated using the same standardized inference procedure were included. In the plot, FPS is shown on the x-axis and mAP@0.75 on the y-axis, the bubble area represents the number of model parameters, and the labels next to the bubbles indicate the GFLOPs and parameter count. The proposed RGC-YOLO model is highlighted with a star.

As shown in **Figure 7**, RGC-YOLO is located in the upper-right region of the localization accuracy–speed trade-off plot and has a relatively small bubble area. Compared with the baseline YOLOv8n model, RGC-YOLO improved mAP@0.75 by 2.65% and substantially reduced the parameter count, while maintaining an essentially unchanged inference speed, indicating that the lightweight design did not cause a noticeable loss of inference efficiency. Meanwhile, the reduction in parameter count did not translate into a corresponding increase in FPS, suggesting that practical inference speed is not determined solely by model size. Compared with YOLO11n, RGC-YOLO achieved higher inference speed and a lower parameter count at the cost of only a small reduction in localization accuracy. Compared with YOLO12n, it maintained comparable localization accuracy while exhibiting a clear speed advantage. Although YOLOv5n achieved a higher FPS, RGC-YOLO provided better high-IoU localization accuracy with fewer parameters. Star-YOLO had the smallest parameter count but showed relatively low inference speed and localization accuracy, further indicating that a smaller parameter count does not necessarily correspond to higher practical inference efficiency. Overall, RGC-YOLO did not achieve an absolute advantage in every individual metric but provided a more favorable trade-off among localization accuracy, inference speed, and model complexity.

### 3.2 Component ablation and structural validation of RGC-YOLO

#### 3.2.1 Overall component ablation study

To evaluate the impact of each improved module on detection performance and model complexity, this study uses YOLOv8n as the baseline model, denoted as B0, and conducts ablation experiments. B1–B4 represent model variants resulting from the incorporation of different improvement modules into B0. The experimental results for each model variant are shown in **Table 5**.

**Table 5:** Ablation experiment results for different module combinations.

ID	R-UIB	G-C2f	DIoU	P (%)	R (%)	mAP@0.5 (%)	mAP@0.75 (%)	mAP@0.5:0.95 (%)	Params (M)	GFLOPs
B0	×	×	×	82.37± 2.60	77.86± 1.05	84.20± 0.76	75.87± 1.37	70.79± 1.32	3.01	8.20
B1	√	×	×	86.99± 2.03	77.02± 0.71	85.27± 0.55	77.66± 1.23	71.51± 0.77	2.23	7.17
B2	×	√	×	86.12± 3.30	78.06± 2.60	85.66± 0.94	79.17± 0.34	73.20± 0.31	2.73	7.97
B3	√	√	×	84.04± 2.19	78.18± 2.29	84.22± 0.90	77.75± 0.35	71.87± 0.47	1.95	6.94
B4	√	√	√	85.83± 0.60	78.44± 0.23	85.51± 1.14	78.52± 0.51	72.42± 0.90	1.95	6.94

As shown in **Table 5**, compared to B0, B1 has a 25.91% reduction in Params and a 12.56% reduction in GFLOPs. At the same time, most of B1’s performance metrics showed improvement, with only the recall rate declining slightly. The results above indicated that the R-UIB module primarily serves to lighten the model’s structure and maintains detection performance well even after reducing the model’s complexity. This is attributed to R-UIB’s simplification of the feature transformation path and the residual propagation path, which enables it to reduce redundant computations when processing deep, high-channel-width features while preserving deep semantic information (Sandler et al., 2018; Qin et al., 2024). Compared to B0, B2 shows a 9.30% reduction in Params and a 2.80% reduction in GFLOPs. Although the reduction in complexity for B2 is smaller than that for B1, its mAP@0.75 has increased by 3.30%, and its mAP@0.5:0.95 has also risen from 70.79% to 73.20%. The results above indicate that the G-C2f module substantially improves detection performance at higher IoU thresholds while introducing only a limited reduction in model complexity. This improvement may be attributed to the lightweight feature-transformation strategy employed at the P5 semantic fusion node, which reduces redundant computations in high-level feature processing while retaining the semantic aggregation capability required for accurate weed localization (Lin et al., 2017a; Han et al., 2020).

In addition, compared to B0, B3 reduces Params by 35.22% and GFLOPs by 15.37%, while maintaining stable overall detection performance. Compared to B1, B3 further reduces the model size while improving the mAP@0.5:0.95 by 0.36%. Compared to B2, B3 has 28.57% fewer parameters and 12.92% fewer GFLOPs, but its mAP@0.5:0.95 is 1.33% lower. This indicates that, although the combination of the R-UIB module and the G-C2f module further reduces model complexity, a typical trade-off between detection performance and model complexity persists as the model becomes more lightweight. This illustrates that, although combining the R-UIB module with the G-C2f module can further reduce model complexity, streamlining both the deep feature extraction and high-level semantic fusion processes simultaneously also weakens the model’s feature representation capabilities. Therefore, while redundant computations are reduced, detection performance also declines to some extent, reflecting the persistent trade-off between detection accuracy and model complexity (Tan and Le, 2019). Compared to B3, B4’s Params and GFLOPs remain unchanged, while mAP@0.75 has increased by 0.77% and mAP@0.5:0.95 by 0.55%. This indicates that DIoU improves localization performance at higher IoU thresholds without increasing model complexity. This improvement is attributed to the incorporation of the normalized center-point distance between the predicted and ground-truth boxes into the bounding box regression objective, which strengthens the geometric constraint on box localization. Since DIoU modifies only the regression loss during training and introduces no additional network layers, the Params and GFLOPs remain unchanged (Zheng et al., 2020).

In summary, the three improvements are not simply a combination of the same feature, but rather act separately on the processes of deep feature extraction, high-level semantic fusion, and bounding box regression. B1 and B2 demonstrate that R-UIB and G-C2f have different performance focuses; B3 illustrates the trade-off between detection performance and model complexity during the joint lightweighting process; and B4 further improves high-IoU localization performance without increasing structural overhead. Accordingly, B4 was identified as the final configuration for RGC-YOLO. Subsequent experiments will further analyze the model from three aspects: budget matching, module placement, and bounding box regression loss.

#### 3.2.2 Budget-matched comparison of the R-UIB module

To determine whether the performance improvements achieved by R-UIB stem from its structural design rather than merely from a reduction in channel width, this study conducted comparative experiments under similar model complexity conditions. Specifically, R0 is the baseline model YOLOv8n, and R1 is a model variant that achieves lightweight processing solely by reducing the channel width. R2 is a model that incorporates R-UIB modules deep within the backbone network to achieve a structured, lightweight restructuring. The overall performance of R0, R1 and R2 are shown in **Table 6**.

**Table 6:** Budget-matched comparison results for the R-UIB module.

ID	F1 (%)	mAP@0.5 (%)	mAP@0.75 (%)	mAP@0.5:0.95 (%)	Params (M)	GFLOPs
R0	80.02± 0.69	84.20± 0.76	75.87± 1.37	70.79± 1.32	3.01	8.20
R1	79.75± 0.28	83.89± 0.63	75.98± 1.46	70.18± 1.28	2.34	7.34
R2	81.68± 0.54	85.27± 0.55	77.66± 1.23	71.51± 0.77	2.23	7.17

As shown in **Table 6**, compared to R0, R1 has 22.26% fewer parameters and 10.49% fewer GFLOPs. However, the mAP@0.5:0.95 fell by 0.61%, and the F1 also declined slightly. This demonstrates that, although simply reducing the channel width can lower the model’s complexity, it also affects overall detection performance to some extent. This is because a reduction in the number of channels limits the model’s ability to transmit and represent feature information, making it difficult for the model to fully retain effective information relevant to object detection within the limited channel capacity (Tan and Le, 2019). Compared to R1, R2 achieved a 1.68% increase in mAP@0.75 and a 1.33% increase in mAP@0.5:0.95, while also improving the F1 score, despite a further reduction in model complexity. Compared to R0, R2 features a 25.91% reduction in parameters and a 12.56% reduction in GFLOPs, while achieving a 1.79% improvement in mAP@0.75 and a 0.72% improvement in mAP@0.5:0.95. The results above indicate that, compared to directly reducing the channel width, the R-UIB module demonstrates better performance retention within a similar model complexity budget. It also improves localization performance at higher IoU thresholds while reducing model complexity, thereby demonstrating the advantage of its structured lightweight design.

Furthermore, the comparison between R1 and R2 indicates that the advantage of R-UIB cannot be attributed solely to parameter reduction. Rather than directly compressing the channel width, R-UIB reorganizes feature transformation in the deep high-channel stages while preserving information propagation through the residual paths, thereby maintaining the representation capacity of high-level semantic features under a comparable computational budget (Sandler et al., 2018; Qin et al., 2024).

#### 3.2.3 Placement ablation of the G-C2f module

To verify the impact of the G-C2f module’s placement on the model’s overall performance, this study conducted location ablation experiments. G0 is the baseline model, which includes only the R-UIB module and does not incorporate the G-C2f module. G1, G2, and G3 represent configurations in which the G-C2f module is placed at the P4-scale feature fusion point, the P4–P5-scale feature fusion point, and the P5-scale feature fusion point, respectively, based on the G0 architecture. The results are shown in **Table 7**.

**Table 7:** Placement ablation results of the G-C2f module.

ID	F1 (%)	mAP@0.5 (%)	mAP@0.75 (%)	mAP@0.5:0.95 (%)	Params (M)	GFLOPs
G0	81.68± 0.54	85.27± 0.55	77.66± 1.23	71.51± 0.77	2.23	7.17
G1	80.55± 0.52	84.85± 0.86	77.58± 0.26	71.32± 0.26	2.16	6.94
G2	81.34± 1.08	84.13± 0.93	76.55± 0.50	70.93± 0.81	1.88	6.72
G3	80.96± 0.38	84.22± 0.90	77.75± 0.35	71.87± 0.47	1.95	6.94

**Table 7** shows that the placement of the G-C2f module has varying degrees of impact on model complexity and detection performance. Compared to G0, G1 shows a 3.14% reduction in Params and a 3.21% reduction in GFLOPs, but all mAP metrics have declined. This indicates that while introducing the G-C2f module at the P4 feature fusion point reduces model complexity, it weakens the neck network’s ability to propagate spatial information from the middle layers. This is attributed to the fact that the P4 features, which have relatively high resolution, retain more spatial and localization information, and this information plays a crucial role in the process of cross-scale feature transmission and fusion (Lin et al., 2017a; Liu et al., 2018). In addition, as compared to G0, G2 reduces the number of parameters by 15.70% and GFLOPs by 6.28%, further reducing the model size. However, G2’s mAP@0.75 dropped by 1.11%, and its mAP@0.5:0.95 also fell from 71.51% to 70.93%. These results indicate that while introducing the G-C2f module at the feature fusion points for both P4 and P5 scales further reduces the model’s complexity, it weakens the neck network’s ability to fuse multiscale features, thereby affecting localization performance at higher IoU thresholds. The reason for this is that feature information from two different levels is compressed simultaneously (Lin et al., 2017a; Liu et al., 2018).

Comparing G3 to G1, the number of parameters in G3 was reduced from 2.16 million to 1.95 million, a decrease of 9.72%, while GFLOPs remained at 6.94, and mAP@0.5:0.95 improved by 0.55%. Compared to G2, G3’s Params increased by only 0.07 M, but the mAP@0.75 and mAP@0.5:0.95 improved by 1.20% and 0.94%, respectively. These results show that introducing the G-C2f module only at the P5-scale feature fusion stage enables high IoU localization performance while maintaining low model complexity.

From the perspective of structural performance, the effectiveness of G-C2f depends on the degree of alignment between its lightweight design approach and the hierarchical properties of its features. The G-C2f module primarily functions by reducing redundant computations in high-channel feature transformations. Since P5 has a higher channel dimension, lower spatial resolution, and more concentrated high-level semantic information, it is better suited as the placement location for the G-C2f module. This is because lightweight feature transformations are better suited for high-level features that have a high degree of channel redundancy and are less dependent on spatial details (Lin et al., 2017a; Han et al., 2020). The experimental results also show that, with G-C2f, more is not necessarily better; the key lies in placing it at high-level semantic fusion nodes that have high computational redundancy and are relatively less dependent on spatial details. After evaluating the detection performance and model complexity of various configuration schemes, this study ultimately adopts G3 as the configuration scheme for the G-C2f module.

### 3.3 Bounding-box regression loss comparison and interpretability analysis

#### 3.3.1 Comparison of bounding-box regression losses

To further analyze the impact of different bounding box regression loss functions on the model’s detection performance, building upon a model architecture that integrates R-UIB and G-C2f, this study conducts loss function ablation experiments by setting the bounding box regression loss to CIoU (Zheng et al., 2020), GIoU (Rezatofighi et al., 2019), SIoU, and DIoU, respectively. CIoU was used as the default bounding box regression loss in the original architecture. The experimental results are shown in **Table 8**.

**Table 8:** Ablation results of different bounding-box regression losses.

Loss function	P (%)	R (%)	F1 (%)	mAP@0.5 (%)	mAP@0.75 (%)	mAP@0.5:0.95 (%)
CIoU	84.04± 2.19	78.18± 2.29	80.96± 0.38	84.22± 0.90	77.75± 0.35	71.87± 0.47
GIoU	84.65± 0.76	78.58± 1.59	81.49± 0.57	85.54± 0.66	77.64± 0.59	72.12± 0.64
SIoU	84.97± 2.32	79.08± 2.15	81.88± 0.24	85.13± 0.72	76.84± 0.67	71.80± 0.49
DIoU	85.83± 0.60	78.44± 0.23	81.97± 0.39	85.51± 1.14	78.52± 0.51	72.42± 0.90

From **Table 8**, it can be seen that compared to the default loss function, CIoU, using DIoU resulted in a 1.79% increase in the model’s P-score, a 0.77% increase in mAP@0.75, and a 0.55% increase in mAP@0.5:0.95. The improvement in high-IoU evaluation metrics indicates that DIoU mainly enhances the localization accuracy of predicted bounding boxes rather than simply increasing the number of detected targets.

A further comparison of the different loss functions shows that although GIoU achieved the highest mAP@0.5, DIoU was only 0.03% lower, while outperforming GIoU in mAP@0.75 and mAP@0.5:0.95 by 0.88% and 0.30%, respectively. Although SIoU achieved the highest R, its mAP@0.75 and mAP@0.5:0.95 were both lower than those of DIoU. This indicates that improving target coverage does not necessarily lead to a direct improvement in localization accuracy. Although SIoU increased the recall rate, the lower high-IoU metrics suggest that some predicted bounding boxes still exhibited insufficient spatial alignment with the actual weed regions.

The above results indicate that, within the RGC-YOLO framework, the bounding-box regression loss mainly affects the optimization of localization errors rather than the overall target recognition capability. This finding is consistent with Zheng et al. (2020), who demonstrated that incorporating spatial relationships between predicted and ground-truth boxes can improve regression efficiency and localization accuracy. Therefore, DIoU achieves a better balance between detection accuracy and localization quality under complex field conditions.

#### 3.3.2 Eigen-CAM-based interpretability analysis

**Figure 8:**
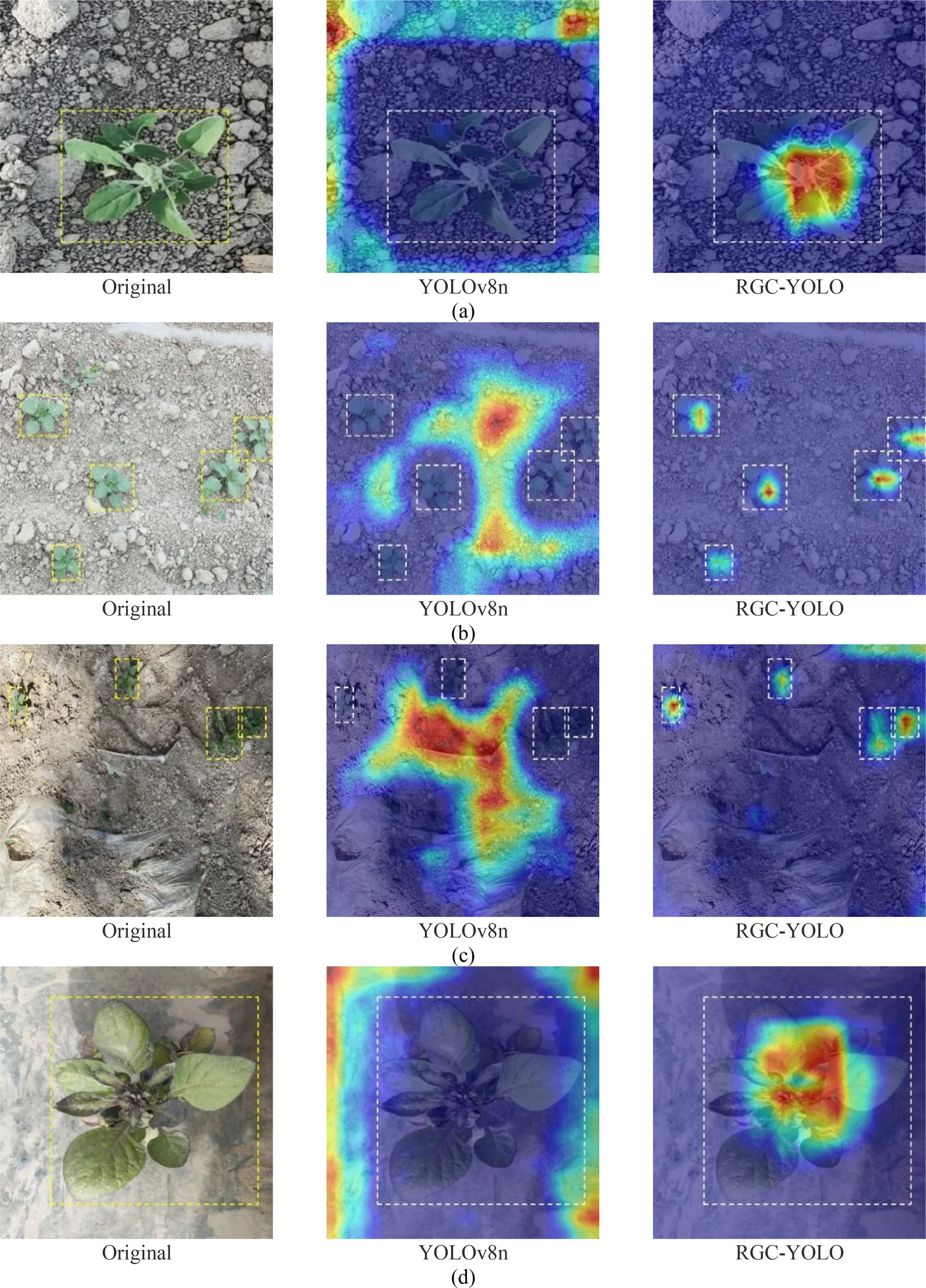
Comparison of Eigen-CAM visualization results. **(a)** Single-target scene. **(b)** Multi-object, small-object scene. **(c)** Complex ground scene. **(d)** Single-target scene.

Eigen-CAM generates response maps by extracting the principal components of the feature representations from convolutional layers, thereby visually illustrating the regions where the model responds to key features in the input image, thus providing a qualitative explanation of the model’s detection behavior (Muhammad and Yeasin, 2020; Bany Muhammad and Yeasin, 2021; Karim et al., 2024). Therefore, this study uses Eigen-CAM to visually compare the deep feature responses of the YOLOv8n baseline model and RGC-YOLO in order to analyze the differences in feature attention between the two models in complex cotton field scenarios, as shown in **Figure 8**.

As seen in **Figure 8**, the feature response regions of YOLOv8n are somewhat scattered in certain scenes, and there is some response in the background areas. In the single-target scenes shown in **Figure 8a** and **Figure 8d**, some of the high-response regions in YOLOv8n are distributed outside the target bounding box, along the image edges, and in the surrounding ground areas, while the responses within the target region are relatively weak. In contrast, RGC-YOLO’s high-response regions are more concentrated on the weed objects and their surrounding areas, and the activation locations show better alignment with the annotated target regions.

In the multi-object, small-object scene shown in **Figure 8b**, the feature responses of YOLOv8n exhibit a distinct diffusion pattern, with some high-response regions located in non-target areas such as soil textures. In contrast, RGC-YOLO produced clearer and more localized activations around multiple weed target regions, whereas the responses in background areas were reduced. In the complex ground scene shown in **Figure 8c**, the response regions of YOLOv8n overlap significantly with soil cracks, plastic mulch, and ground textures, whereas the response regions of RGC-YOLO are more concentrated near the annotated targets, indicating that it exhibits more focused target-related feature responses in complex backgrounds. The enhanced feature response localization capability of RGC-YOLO may be attributed to the collaborative optimization of the proposed modules. These structural optimizations enable the model to extract more discriminative weed features in complex cotton field environments and reduce the interference of irrelevant background information on feature responses.

Building on the visual analysis of the representative samples described above, and to further quantify the overall performance of the differences in response distributions shown in **Figure 8** across the positive samples in the test set, this study conducted a quantitative evaluation of the Eigen-CAM responses of YOLOv8n and RGC-YOLO. To ensure comparability between the two models, the quantitative analysis adopted the response extraction settings shown in **Figure 8**. The results of the quantitative evaluation are presented in **Table 9**.

**Table 9:** Quantitative evaluation of Eigen-CAM response localization consistency.

Model/statistic	EBPG (%)	PG accuracy (%)
YOLOv8n	19.68	20.33
RGC-YOLO	27.37	31.71
Paired difference	7.69	11.38
95% CI lower bound	4.29	5.69
95% CI upper bound	11.03	17.07

**Table 9** shows that RGC-YOLO’s EBPG improved from 19.68% for YOLOv8n to 27.37%, whilst its PG accuracy rose from 20.33% to 31.71%. Two indicators show trends suggesting that the improvements in RGC-YOLO are evident at both the overall response energy distribution and peak localization levels. Further analysis shows that the paired differences for EBPG and PG are 7.69% and 11.38%, respectively, with corresponding 95% confidence intervals of 4.29%–11.03% and 5.69%–17.07%, neither of which crosses zero. It demonstrates that, under identical image conditions, the positive changes observed in RGC-YOLO relative to YOLOv8n remain stable in paired comparisons of positive samples on the test set. These quantitative findings are corroborated by the differences in response distributions observed in the representative samples shown in **Figure 8**, indicating that the responses at the feature layer of RGC-YOLO exhibit a higher degree of spatial correspondence with the actual weed regions, whilst relatively reducing the interference of non-target background regions on the response distributions. These findings demonstrate that RGC-YOLO achieves a more target-oriented feature representation by suppressing irrelevant background activations, providing further evidence of its enhanced detection robustness in complex cotton field environments.

### 3.4 Edge deployment and external validation

#### 3.4.1 Edge deployment evaluation on the Jetson AGX Orin platform

To evaluate the edge deployment adaptability of RGC-YOLO for cotton-field weed detection, the trained weights of YOLOv8n, YOLO11n, YOLO12n, and RGC-YOLO were deployed on the NVIDIA Jetson AGX Orin platform, and FP32, FP16, and INT8 inference engines were constructed using TensorRT 10.3.0. Among these, YOLOv8n serves as the baseline model for RGC-YOLO, while YOLO11n and YOLO12n demonstrated strong overall performance in the aforementioned comparative experiments; therefore, they are used as reference models for comparison. Deployment performance is evaluated based on three criteria: detection accuracy, storage overhead, and inference efficiency. Detection accuracy was evaluated using mAP@0.5, mAP@0.75, and mAP@0.5:0.95, storage overhead was characterized by inference engine size, and inference efficiency was assessed in terms of latency and throughput (FPS). For the inference-efficiency evaluation, all models were benchmarked under the same settings using TensorRT’s native trtexec tool, with an input size of 640 × 640 and a batch size of 1. A 1000-ms warm-up was followed by a 60-s measurement period. The detailed results are shown in **Table 10**.

**Table 10:** Edge deployment performance comparison of lightweight YOLO models on the Jetson AGX Orin platform.

Inference format	Model	mAP@0.5 (%)	mAP@0.75 (%)	mAP@0.5:0.95 (%)	Engine size (MB)	Latency (ms)	FPS
FP32	YOLOv8n	82.54	72.92	68.23	13.07	3.06	360.2
	YOLO11n	82.45	78.19	71.18	11.66	3.40	323.2
	YOLO12n	83.84	76.83	71.40	12.65	5.21	203.9
	RGC-YOLO	84.17	77.36	71.39	9.13	3.05	364.5
FP16	YOLOv8n	82.58	72.63	68.04	8.52	1.91	596.8
	YOLO11n	82.46	78.19	71.15	7.98	2.02	564.1
	YOLO12n	83.85	76.88	71.40	8.60	3.18	341.0
	RGC-YOLO	84.15	77.32	71.22	6.18	1.83	630.5
INT8	YOLOv8n	76.44	67.06	62.08	4.80	1.47	790.1
	YOLO11n	80.03	74.53	67.30	5.29	1.73	651.6
	YOLO12n	80.95	73.27	67.95	5.50	3.54	299.0
	RGC-YOLO	78.88	70.39	64.10	3.60	1.46	800.7

As shown in **Table 10**, the lightweight architecture of RGC-YOLO effectively translates into edge-deployment benefits on the Jetson AGX Orin platform. Compared with the baseline model YOLOv8n, RGC-YOLO achieved smaller inference engine sizes across FP32, FP16, and INT8 precision formats while maintaining higher detection accuracy. Specifically, in the FP16 precision format, RGC-YOLO improved mAP@0.5:0.95 by 3.18% and reduced the inference engine size by 27.46%, while achieving lower inference latency and higher throughput. The results show that RGC-YOLO’s lightweight design effectively reduces storage overhead and improves inference efficiency on edge devices.

A further comparison with YOLO11n and YOLO12n shows that RGC-YOLO maintained competitive detection performance under both FP32 and FP16 precision formats, while achieving the smallest inference engine size, the lowest inference latency, and the highest throughput among the four models. Taking the FP16 precision format as an example, RGC-YOLO achieved an mAP@0.5:0.95 of 71.22%, which was only 0.18% lower than that of YOLO12n, while reducing the inference engine size and inference latency by 28.14% and 42.45%, respectively. This indicates that RGC-YOLO has strong edge deployment competitiveness compared with the reference models.

It is worth noting that the changes in inference efficiency after INT8 quantization vary across different models. YOLOv8n, YOLO11n, and RGC-YOLO achieved lower inference latency in the INT8 format, while YOLO12n’s inference latency increased from 3.18 ms in the FP16 format to 3.54 ms, and its throughput decreased from 341.0 FPS to 299.0 FPS. Previous studies have indicated that the actual inference efficiency after low-precision quantization depends not only on numerical precision, but is also closely related to the model architecture, operator types, requantization overhead, and execution paths on the target hardware (Shi et al., 2024; Liu et al., 2025; Song et al., 2025; Zhao et al., 2026). YOLO12n adopts a network design centered on attention mechanisms (Tian et al., 2026), and the performance variation of its INT8 engine on this platform further demonstrates that reducing numerical precision does not necessarily translate into higher practical inference efficiency.

For RGC-YOLO, after converting from FP32 to FP16, its mAP@0.5:0.95 decreased by only 0.17%, while the inference engine file size and inference latency were reduced by 32.31% and 40.00%, respectively. When further quantizing from FP16 to INT8, the inference engine file size and inference latency were reduced by 41.75% and 20.22%, respectively, but the mAP@0.5:0.95 decreased by 7.12%. This indicates that INT8 quantization can further reduce the model’s memory overhead and inference latency, but results in a noticeable loss in detection accuracy. Therefore, in application scenarios with stricter resource constraints, INT8 can be used as an alternative deployment format. Considering detection accuracy, storage overhead, and inference efficiency, FP16 achieved a better balance among the evaluation metrics and can be used as the preferred deployment format for RGC-YOLO on the Jetson AGX Orin platform.

**Figure 9:**
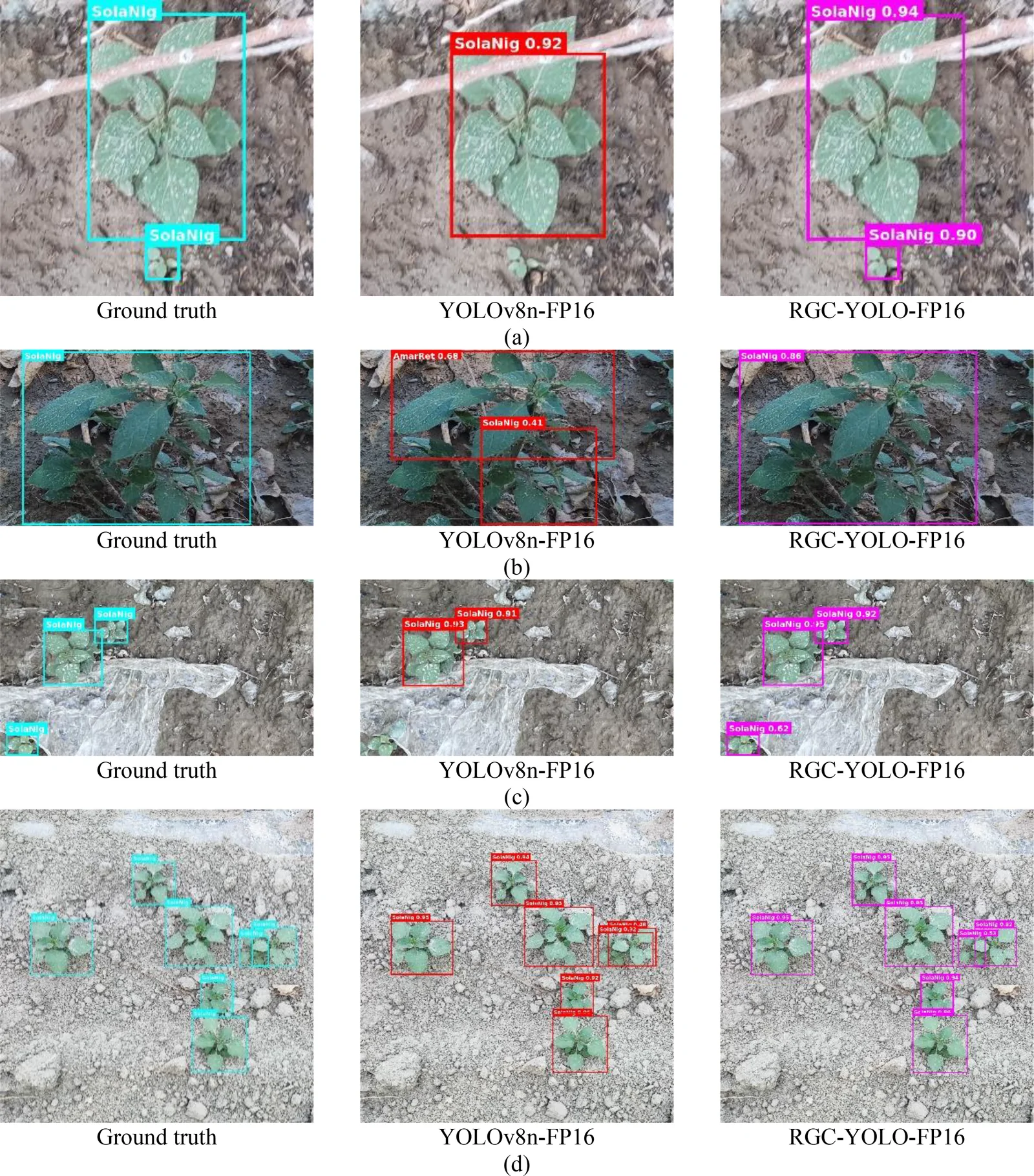
Comparison of inference performance on the Jetson AGX Orin platform. **(a–d)** Representative images from the test set.

To further demonstrate the practical edge-side inference performance, inference was performed on representative images from the test set using the FP16 TensorRT engine on the Jetson AGX Orin platform. The results are shown in **Figure 9**.

In summary, RGC-YOLO demonstrates strong adaptability for edge deployment on the Jetson AGX Orin platform. Compared with the baseline model YOLOv8n and the reference models YOLO11n and YOLO12n, RGC-YOLO showed overall advantages in detection accuracy, inference engine size, inference latency, and throughput. The FP16 format substantially reduces storage overhead and latency with minimal loss of precision, making it the preferred deployment option. In scenarios with stricter resource constraints, INT8 quantization can still serve as an alternative. The inference results in **Figure 9** further provide an intuitive demonstration of the edge-side detection performance of RGC-YOLO, offering a reliable reference for subsequent intelligent cotton-field weed detection applications.

#### 3.4.2 External validation on public dataset

**Figure 10:**
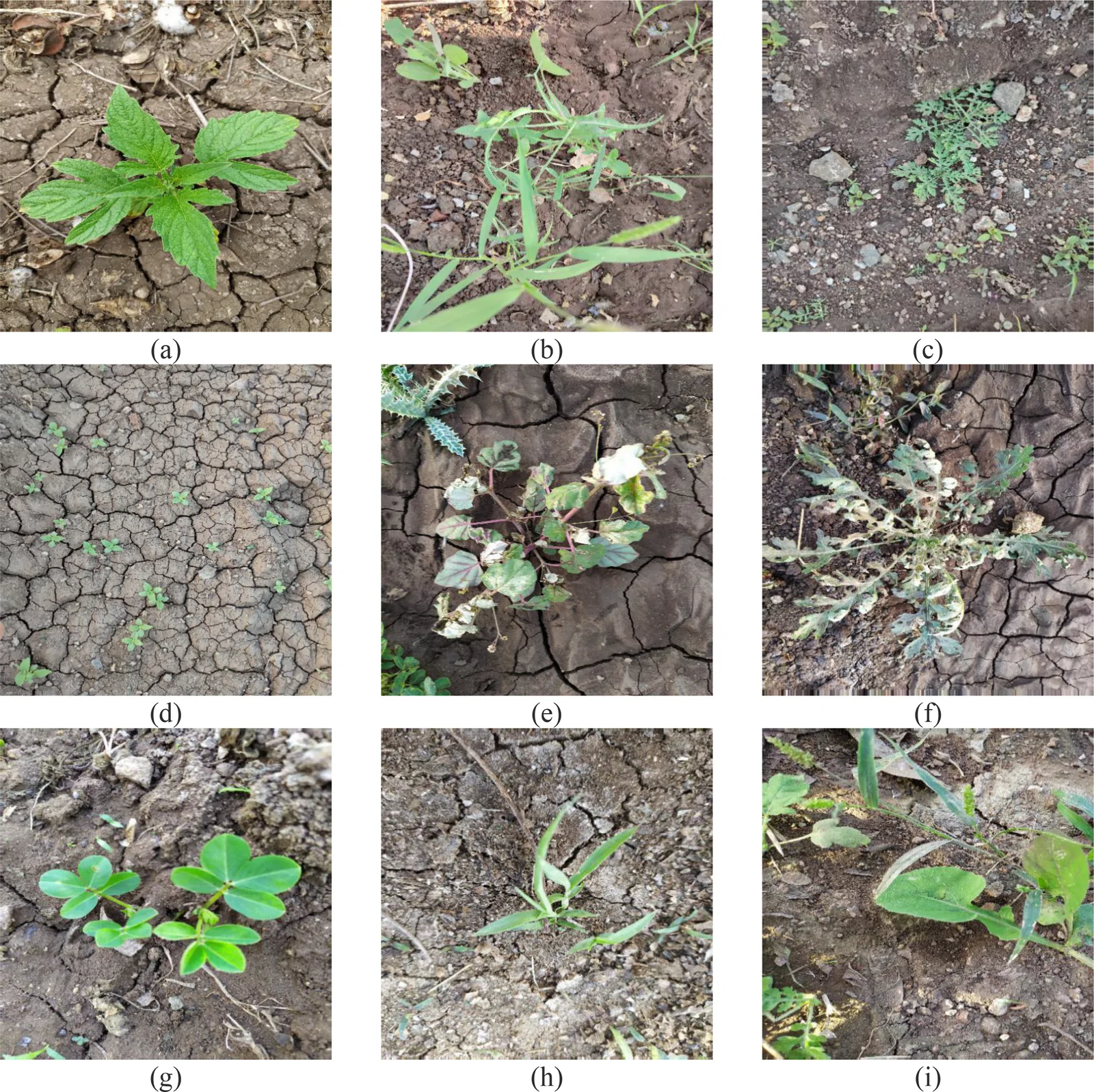
Representative images from the external crop–weed detection dataset. **(a–i)** Representative images from the dataset. Source: Crop and Weed Detection Data with Bounding Boxes, Kaggle; CC0: Public Domain.

To examine the generalization adaptability of RGC-YOLO in external agricultural scenarios, a public dataset was introduced for supplementary validation in this study. This dataset is designed for crop and weed detection tasks in sesame fields and includes two categories, namely crop and weed. Representative images are shown in **Figure 10**.

The baseline model YOLOv8n was selected for comparison with RGC-YOLO under the same data split, training parameters, and evaluation procedure, and the results are shown in **Table 11**.

**Table 11:** External validation results on a binary crop–weed detection dataset.

Model	P (%)	R (%)	F1 (%)	mAP@0.5 (%)	mAP@0.75 (%)	mAP@0.5:0.95 (%)	Params (M)	GFLOPs
YOLOv8n	77.10± 1.83	76.32± 1.75	76.71± 0.29	79.92± 0.13	55.03± 1.61	50.35± 0.41	3.01	8.20
RGC-YOLO	82.41± 1.45	74.71± 1.13	78.37± 0.29	82.20± 0.36	56.70± 1.09	52.41± 0.49	1.95	6.94

The results in **Table 11** show that, compared with YOLOv8n, RGC-YOLO improved mAP@0.5:0.95 by 2.06%, while reducing the number of parameters and GFLOPs by 35.22% and 15.37%, respectively. Although its recall decreased by 1.61%, its F1 score improved by 1.66%, indicating that the model achieved a better overall trade-off between precision and recall. These results demonstrate that RGC-YOLO maintained favorable detection performance in the external binary crop–weed detection scenario while retaining its lightweight advantage, demonstrating a certain degree of cross-scenario robustness. The results also indicate that RGC-YOLO’s lightweight structural optimization does not rely excessively on specific visual features in the original cotton field data, demonstrating its stability under various crop background conditions.

In this study, to further evaluate the detection performance of RGC-YOLO on different datasets, we introduced the publicly available multi-class weed dataset 2SeasonWeedDet8 for external validation. This dataset includes eight weed species: waterhemp, carpetweed, morning glory, goosegrass, spotted spurge, Palmer amaranth, purslane, and ragweed. The resulting data are shown in **Table 12**.

**Table 12:** External validation results on the public multi-class weed dataset.

Model	P (%)	R (%)	mAP@0.5 (%)	mAP@0.75 (%)	mAP@0.5:0.95 (%)	Params (M)	GFLOPs
YOLOv8n	93.94± 0.17	89.02± 0.80	95.39± 0.13	92.72± 0.32	89.51± 0.78	3.01	8.20
RGC-YOLO	92.62± 1.17	89.40± 0.88	95.40± 0.09	92.53± 0.26	89.31± 0.18	1.95	6.94

**Table 12** shows that RGC-YOLO maintains overall detection performance on the 2SeasonWeedDet8 dataset that is essentially on par with that of YOLOv8n. In particular, Recall improved by 0.38%, and the differences in all three mAP metrics compared to YOLOv8n were no greater than 0.20%. At the same time, RGC-YOLO’s number of parameters and GFLOPs were reduced by 35.22% and 15.37%, respectively. The above results indicate that, despite a significant reduction in model complexity, RGC-YOLO is still able to maintain detection performance comparable to that of YOLOv8n across different weed categories. In summary, RGC-YOLO strikes a good balance between detection performance and model efficiency, supporting its application in various agricultural weed detection tasks. Together with the edge-deployment results presented in Section 3.4.1, these findings further indicate the potential of RGC-YOLO as an edge-side visual perception model for site-specific weed management in cotton fields. Previous cotton-field studies have demonstrated the applicability of lightweight weed detection on mobile platforms (Fan et al., 2024) and its integration with downstream weed-targeting systems (Karim et al., 2024).

## 4 Conclusions

This study addresses the need for lightweight weed detection in the complex cotton field environments of Xinjiang. We constructed a cotton field weed detection dataset and proposed the RGC-YOLO model. Through a systematic evaluation of the model’s overall performance and applicability, the following main conclusions can be drawn:

1. RGC-YOLO achieves a good balance between accuracy and computational complexity in the task of detecting weeds in complex cotton fields. Compared with YOLOv8n, RGC-YOLO improved Precision, mAP@0.75, and mAP@0.5:0.95 by 3.46%, 2.65%, and 1.63%, respectively, while reducing the number of parameters and GFLOPs by 35.22% and 15.37%, respectively. These results indicate that the proposed model effectively reduced model complexity while improving localization performance under a higher IoU threshold.
2. R-UIB and G-C2f constituted the main lightweight structures of RGC-YOLO. The ablation results showed that, compared with direct channel-width reduction, R-UIB maintained better detection performance while reducing model complexity. Moreover, placing G-C2f at the P5 feature fusion point achieved a more favorable balance among deep semantic feature fusion, high-IoU localization performance, and model complexity.
3. The DIoU loss function further improved bounding-box localization performance under the lightweight structure. Compared with other bounding-box regression losses, DIoU showed more balanced performance in Precision, F1, and metrics related to higher IoU thresholds. Furthermore, Eigen-CAM visualization and quantitative analysis demonstrated that RGC-YOLO generated more target-oriented feature responses in weed regions while reducing irrelevant background activations, providing interpretability evidence for its improved detection performance.
4. RGC-YOLO showed good adaptability to edge deployment and external datasets. Tests on the Jetson AGX Orin platform indicated that the FP16 TensorRT format achieved a good overall balance among detection accuracy, inference speed, and storage overhead, making it the preferred deployment format. External validation on a sesame-field binary crop–weed detection dataset and the 2SeasonWeedDet8 multi-class weed dataset showed that RGC-YOLO maintained comparable detection performance to YOLOv8n while preserving its lightweight advantage.

The current results have demonstrated the detection performance, cross-dataset adaptability, and edge-deployment feasibility of RGC-YOLO on the self-constructed cotton-field weed dataset, two external datasets, and the Jetson AGX Orin platform. Building on these results, future work will broaden data collection across additional geographical regions, weed species, and imaging conditions, extend model evaluation to additional edge devices, and further assess the real-time performance and operational stability of RGC-YOLO under continuous field operating conditions. To reduce the dependence of supervised detection models on predefined categories and annotated data, future research will also investigate the application of vision–language models to zero-shot weed detection and visual reasoning in complex field scenes, thereby improving adaptability to unseen weed categories and open scenarios.

## Data Availability

The raw data supporting the conclusions of this article will be made available by the authors, without undue reservation.
